# Supplementary material for: Leukocyte inflammatory phenotype and function in migraine patients compared with matched non-migraine volunteers: a pilot study
Source: BMC Neurol. 2022 Jul 27;22:278. doi: 10.1186/s12883-022-02781-4 (PMC9327171; doi:10.1186/s12883-022-02781-4)
Supplement: Supplementary file 1 — Additional file 1. Supplemental Table 1.1 Means, standard deviations, and medians for groups defined by control and migraine status, sub-setting by chronic vs episodic and medication overuse. Supplemental Table 1.2 Means, standard deviations, and medians for groups defined by control and migraine status, sub-setting by chronic vs episodic and medication overuse. Supplemental Table 1.3 Means, standard deviations, and medians for groups defined by control and migraine status, sub-setting by chronic vs episodic and medication overuse. [file 12883_2022_2781_MOESM1_ESM.zip › 12883_2022_2781_ReferencePDF.pdf]

ACCEPTED MANUSCRIPT

Leukocyte inflammatory phenotype and function in migraine patients compared with matched non-migraine volunteers: A pilot study

Hongtao Li[1, 4*], Qiang Fu[2*], Kamaira Philips[3], Yufei Sun[2], Keturah R. Faurot[4], Susan A. Gaylord[4], John Douglas Mann[4,5]

1 Department of Science and Mathematics, Saint Mary of the Woods College, Indiana, USA

2 School of Pharmacology, Institute of Aging Medicine, Binzhou Medical University, Yantai 264003, PR China

3 Division of Comprehensive Oral Health, Adams School of Dentistry, University of North Carolina at Chapel Hill, North Carolina, USA

4 Program on Integrative Medicine, Department of Physical Medicine and Rehabilitation, University of North Carolina at Chapel Hill, North Carolina, USA

5 Department of Neurology, University of North Carolina at Chapel Hill, North Carolina, USA

Accepted manuscript

*Both authors contributed equally to this work.

Address correspondence to: Dr. Hongtao Li, Hulman Hall Room116, Department of Science and Mathematics, Saint Mary-of-the-Woods College, 1 St Mary of Woods Coll, IN 47876, USA; Phone: 812-223-1027 Email: hongtaoli66@gmail.com

Running title: Leukocyte phenotype and function, inflammation and migraine

Summary sentence: Migraineurs' peripheral blood shows altered monocyte and T cell subpopulation compared with the control group, suggesting migraine is potentially associated with inflammation and autoimmune processes.

ACCEPTED MANUSCRIPT

ABSTRACT

Background: Migraine is a neurological condition characterized by chronic inflammation. However, not much is known about the potential role of peripheral blood immune cells in the pathophysiology of migraine.

Methods: We investigated the status of peripheral blood immune cells of 15 adults with frequent episodic or chronic migraine recruited chronologically from a randomized clinical trial (RCT) on Nutrition for Migraine (NCCIH 5R01AT007813-05) and 15 non-migraine, healthy volunteers (control) matched by age, gender, and Body Mass Index (BMI).

Continuous variables were presented as means ± standard deviationas well as medians, and comparisons between patients and healthy volunteers were performed with non-parametric Wilcoxon signed rank tests. Statistical analysis was performed using Stata (StataCorp. 2019. Stata Statistical Software). Fluorescence-Activated Cell Sorting (FACS) data were processed using FlowJo software (Ashland, OR: Becton, Dickenson and Company; 2019).

Results: We observed that migraineurs had a significantly lower percentage of non-classical monocytes ($CD14^{+}CD16^{++}$) in blood circulation, compared to the control group. In addition, Migraineurs also showed a significantly lower

Accepted manuscript

ACCEPTED MANUSCRIPT

percentage of blood $CD3^+CD4^+$ helper T cells and $CD4^+CD25^+$ regulatory T cells, compared to controls. Differences in leukocyte surface markers between chronic migraine patients and their matched controls were more prominent than those between episodic migraine patients and their matched controls.

Conclusions: Our results suggest that migraine is associated with dysregulated peripheral immune homeostasis and that inflammation and autoimmunity may play a role in its pathophysiology.

Accepted manuscript

Keywords:

Migraine, inflammation, autoimmunity, Monocytes, T cells

ACCEPTED MANUSCRIPT

Background

Migraine is a major public health problem afflicting over 16% of women, 7% of men, and 12% of the total adult US population and costing billions in health care utilization [1, 2]. Inflammation has long been considered a risk factor in migraine pathogenesis [3, 4]. Examples of elevated inflammatory biomarkers, including fibrinogen and C reactive protein (CRP), have been reported in migraine pathology [5-7]. In 2001, Kemper et al. reviewed 45 clinical studies from 1966 to 1999 and found differences in serum levels of complement, immunoglobulin, histamine, cytokines, and immune cells (monocyte and polymorphonuclear leukocytes) between migraineurs and volunteers without migraine. However, the findings in the various studies were inconsistent and conflicting with each other in many cases [8, 9], indicating a need for further investigation into the role of immune system dysfunction in migraineurs.

Accepted manuscript

Given this emerging link between inflammation and migraine pathogenesis, identification of leukocyte surface antigens could potentially serve as biomarkers to help with diagnosis. In 2006, Du and colleagues reported that the genes significantly up-regulated by migraine were mostly from

platelet/monocytes, while others were from PMNs, $CD4^+$, $CD8^+$ T cells, and NK cells [10]. Research has also shown that lymphotoxin alpha (or tumor necrosis factor-beta) and α-fodrin are among the seven-upregulated genes in migraine with aura, compared with healthy controls [11]. Recently, a genomic-wide analysis using whole blood of 83 migraine cases and 83 age and gender-matched non-migraine controls revealed that multiple immune-inflammatory pathways, such as functional categories of HECS, Microglia, RACTOME, and Gene ontology biological processes, were underlying the pathophysiology of the disorder [12].

Accepted manuscript

Arumugam and Parthasarathy (2016) studied the autoimmune biomarkers $CD4^+CD25^+$ population, helper and suppressor T cell populations, observing a significantly higher $CD4^+$population and lower $CD8^+$T cell population in migraineurs, compared to healthy volunteers. Furthermore, the $CD4^+CD25^+$ population was significantly lower in migraine patients compared to healthy volunteers [13], suggesting that migraine may be related to autoimmunity.

Monocytes comprise a heterogeneous population that plays a vital role in immune surveillance of the central nervous system (CNS). There are at least three different subsets: classical monocytes ($CD14^{++}CD16^-$), intermediate monocytes ($CD14^{++}CD16^+$) and non-classical monocytes ($CD14^+CD16^{++}$)[14]. Waschbisch and colleagues reported that monocytes expressing FcγRIII, or $CD16^+$, which include both intermediate and non-classical monocytes together, were reduced in the peripheral blood and

ACCEPTED MANUSCRIPT

migrated to the sites of inflammation, contributing to the injury of the blood-brain barrier (BBB) in CNS autoimmune diseases such as multiple sclerosis (MS) [15]. The research literature has reported the patrolling behavior of the inflammatory non-classical monocyte subset [16-19] and the activation of genes associated with cytoskeleton mobility [16]. $CD16^{+}$ monocytes have been reported migrating to the central nervous system in MS, HIV associated neurocognitive disorder, and giant cell arteritis [15, 20, 21]. To our knowledge, there is no report showing that $CD16^{+}$ monocytes could migrate to the CNS, thereby decreasing the population percentage in migraineurs' peripheral blood.

Accepted manuscript

T cell surface integrins can play an important role in cellular adhesion to extracellular matrix and cell signaling. Lymphocyte function-associated antigen 1 (LFA-1) and very late antigen-4 (VLA-4) changes have been linked to migraine attacks [24, 25]. LFA-1 is an integrin and belongs to the integrin superfamily of adhesion molecules. It has been reported that T cell surface β2 subunit or CD18 is required for trafficking to the intestinal tissue during an intestinal immune reponses [22].

The objective of the present study was to characterize migraineurs' peripheral leukocyte surface biomarkers and ex vivo functions to determine whether our findings indicate that migraine is related to neuroinflammation and autoimmunity. We used pre-intervention/baseline samples collected from a parent RCTentitled "Clinical and Metabolic Effects of Altering Omega-

ACCEPTED MANUSCRIPT

3 and Omega-6 Fatty Acids in Migraine" funded by an NIH R01-AT007813 (2013-2018) awarded to investigators at the University of North Carolina (UNC) at Chapel Hill [2]. We proposed to examine the leukocyte phenotype profile and cell type function between matched non-migraine volunteers and the migraineurs' baseline data. This comparison may provide mechanistic insights and initial therapeutic strategies for targeting inflammation in migraine.

Accepted manuscript

ACCEPTED MANUSCRIPT

MATERIALS AND METHODS

Participants and ethical clearance

Fifteen consecutive migraine patients who entered the parent R01 RCT study during 2015 and 2016 were selected to be involved in this study. Both studies received approval from UNC's Institutional Review Board (IRB) and the Office of Human Research Ethics and all participants provided informed consent. These subjects were matched with fifteen healthy controls who were recruited to the study from the same population from which the migraineurs were drawn, through university advertising (e.g., mass email). Ascertained via telephone screening and health history questionnaire, eligible volunteers were 18 years of age or older; without chronic pain, pregnancy, or major medical/psychiatric illness; non-smokers; not taking omega-3 supplements; and without concurrent illness. Volunteers were matched to migraine participants by age (10-year categories), sex, and Body Mass Index (BMI) (<20, 20-24.9, 25-29.9, 30-34.9, >35). Migraine patients provided samples on the day of randomization before receiving the intervention. Both migraine patients and control group volunteers had fasted for 8 or more hours prior to phlebotomy. Samples were processed immediately.

Accepted manuscript

Peripheral whole blood staining method using BD Bioscience protocol

Following BD whole blood staining protocols [23], we chose three panels of antibodies, with each panel containing eight antibodies. Table 1 shows the experimental design with the intention to investigate different peripheral leukocyte populations. The antibodies used in this study included: anti-CD3 (APC-H7), anti-CD36 (PerCP-Cy5.5), anti-CD14 (V500), anti-CD16 (BV421), anti-HLA-DR (BB515), anti-CD56 (PE –Cy7), anti-CD86 (APC) (*BioLegend, San Diego, CA*), anti-CD163 (PE) (*BioLegend, San Diego, CA*), anti-CD8 (v500), anti-CD4 (PerCP-Cy5.5) (*BioLegend, San Diego, CA*), anti-CD25 (BB515), anti-CD18 (PE), anti-CD49d (APC), anti-CD11c (BV510), anti-CD123 (BV421), anti-CD19 (PE-Cy7), and anti-CD80 (PE). All antibodies were purchased from BD Bioscience except where stated otherwise. Briefly, appropriate volumes of fluorochrome-conjugated antibody were added to 100 µL of whole blood and incubated 15 to 30 minutes at room temperature. Then, the erythrocytes were lysed, and the samples were washed, fixed, and analyzed within about 24 hours. Data were collected with FACS CANTO II (BD Biosciences) and analyzed using FlowJo software (*Tree Star, Inc., Ashland, OR*).

Accepted manuscript

Peripheral blood mononuclear cells (PBMC) isolation, stimulation, and supernatant collection

PBMCs were isolated from freshly collected human blood samples following density gradient centrifugation over Ficoll-Hypaque density gradient. Briefly, a diluted cell suspension was carefully layered over Ficoll-Hypaque in a conical tube without disturbing the interface, centrifuged at 1500 rpm for 30

ACCEPTED MANUSCRIPT

minutes at room temperature with the brake off. The mononuclear cell layer was at the interphase. The RBC and platelets were washed and a cell counting procedure was performed by TC20™ Automated Cell Counter (BIO-RAD, Hercules, CA).

In vitro stimulations of one million PBMCs per well with 100ng/ml lipopolysaccharide (LPS) for 24 hours were set up in a 6-well culture plate. After stimulation, supernatants were collected for cytokines analysis.

Multiplex measurement of cytokines

To investigate the cytokine production by ex vivo LPS, nine cytokine (TNF-α, IL-6, IL-10, IL-1β, IFN-γ, IL-4, IL-17a, IL-12 p70, and IL-21) levels in PBMC supernatants from pre- and post-LPS 24 hours of stimulations were analyzed using R&D multiplex analysis system at the university's Cytokine & Biomarker Analysis Facility Center.

Cell gating strategies

For monocyte gating, neutrophils, NK cells, B cells, and T cells were successively excluded following the strategy by Mukherjee et al [19]. For T cell gating, subsets were chosen based on conventional bivariate scatterplots of side scatter signal and $CD3^+$, $CD4^+$ T cells, $CD8^+$ T cells, and $CD4^+CD25^+$ T cells subsets.

Statistical analysis

Accepted manuscript

This study presented continuous variables as means ± standard deviations and medians. Due to the small sample sizes, comparisons between patients and healthy volunteers were performed with Wilcoxon signed rank tests. An exploratory sensitivity analysis was undertaken using a hierarchical linear model using random effects of the matched pair. For distributions that were non-normal, estimates were checked using transformations. If interpretation of the results did not change, the original model results were reported. Results are provided for unadjusted differences in means between groups as well as estimates adjusted for chronic migraine and meeting criteria for medication overuse headache. Our statistical analysis was performed using Stata (*StataCorp. 2019. Stata Statistical Software: Release 16. College Station, TX: StataCorp LLC.) .* Fluorescence-Activated Cell Sorting (FACS) data were processed using FlowJo software (*Ashland, OR: Becton, Dickenson and Company; 2019*). Cytokine multiplex data were processed using Stata (*StataCorp. 2017. Stata Statistical Software: Release 15. College Station, TX: StataCorp LLC.) .*

Accepted manuscript

ACCEPTED MANUSCRIPT

RESULTS

Descriptive statistics

A total of 30 subjects -- 15 migraine patients and 15 matched control individuals -- participated in this study. Their demographic comparisons are listed in Table 2. As expected, 80% were female, and the mean (±standard deviation) age was 39.3 ± 12.2 in the migraine sample, and 39.7±12.2 in the controls. BMI means were 27.2±4.6 and 27.4±4.8 in migraine and control groups, respectively.

Accepted manuscript

Migraine has many comorbidities, and people with migraines were significantly more likely to report comorbidities related to inflammation among other conditions [24]. The 15 migraine patients' pain information, including headache, comorbid pain, with or without aura, whether it meets chronic migraine criteria or not, and medication overuse information are provided in the Table 3. The number of migraines in 30 days was 11.2±5.7 days, and the number of headaches in 30 days was 18.6±7.7 days. About 53% of the patients met chronic migraine criteria, based on the most recent diagnostic criteria (ICHD-3), i.e., headache occurring on 15 or more days/month for more than three months, which, on at least eight days/month, has the features of migraine headache. Eight out of 15 patients (53%) met the criteria for overuse of any pain medication.

Comparison of $CD14^{+}CD16^{++}$ monocytes and $CD14^{++}CD16^{-}$ monocytes

ACCEPTED MANUSCRIPT

To examine whether migraine patients have altered blood monocytes, we analyzed the peripheral blood classic and non-classic monocytes in migraineurs and matched control group using flow cytometry. In PBMCs single cells population, we removed NK and T cells, and then divided the HLA-DR+ population into three subgroups and analyzed by CD14 and CD16 expression strength (Fig. 1A). We found that the non-classical monocyte percentage (11.9±10.5)) (Table 4 and Fig. 1B) was lower in the migraine group compared with the control group, (33.9±21.4) ($p=0.005$). In contrast, the percentage of classical monocytes ($CD14^{++}CD16^{-}$) was 49.9±27.6% in the control group and 72.3.±18.1 % in the migraine group ($p=0.06$) (Table 4 and Fig. 1B). Supplemental Table 1 also provides estimates comparing individuals with chronic migraine and medication overuse headache (MOH) to controls. These estimates should be interpreted with caution due to the extremely small sample sizes.

In exploratory sensitivity analyses using hierarchical linear models, we found significant differences at the $p=0.05$ level between patients and controls comparing non-classical (mean difference[MD] -21.65; 95% confidence interval [CI] (-38.88, -4.42)) and classical monocytes (MD26.5; 95% CI: (3.04, 50.12)) in models adjusted for chronic migraine and MOH (Supplemental Table 2).

Percentage of $CD4^{+}$ and $CD4^{+}CD25^{+}$ T cells in blood

Accepted manuscript

ACCEPTED MANUSCRIPT

In our migraine group, CD4+ T cells were significantly lower(64±5.45 vs. 70.6±8.92) ($p=0.035$) and CD8+ T cells were slightly higher ($p=0.3$) in the migraine group compared with the control group (Table 4 and Figure 2). However, the CD4+/CD8 ratio difference is not significant ($p=0.09$). (Table 4).

We identified the $CD4^+CD25^+$ population in this study and display the results in Figure 3A. Compared with the control group, the migraine group had a significantly lower percentage of $CD4^+CD25^+$ T cells (Table 4 and Figure 3B). $CD4^+CD25^+$ T cell percentages in matched control and migraine groups were about 8.3% and 5.7% of $CD3^+$ T cells respectively ($p=0.001$). Significant differences between groups persisted after adjustment for chronic migraine and MOH (MD -2.52; 95% CI: -4.46, -0.57)(Supplemental Table 2).

Accepted manuscript

Integrin CD18 in $CD4^+$ and $CD8^+$ T cells

Compared with the matched control group, migraine patients' CD18, which is part of the LFA-1, were lower in expression levels measured by mean fluorescence intensity (MFI) on both CD4 T and CD8 T cells, as shown in Table 4 and Figure 4. The CD18 MFI on CD4 helper T cells in matched control and migraine group were 735±139 and 619±135, respectively ($p=0.06$). The CD18 MFI on CD8 killer T cells in matched control and migraine groups were 924±239 and 771±250, respectively ($p=0.09$)(Table 4 and Figure 4). Adjusting for chronic migraine and MOH attenuated the differences between the two groups. We found no significant difference in peripheral blood

ACCEPTED MANUSCRIPT

CD49d expression between migraineurs and healthy controls and controlling for chronic migraine and MOH reversed the direction of the difference. (Table 4 and Supplemental Table 1.)

Cytokine analysis after ex vivo stimulation

We stimulated PBMCs from the recruited patients and control volunteers ex. vivo with LPS; nine cytokines from supernatant were measured using the R&D multiplex system. Out of the nine cytokines (TNF-α, IL-6, IL-10, IL-1β, IFN-y, IL-4, IL-17a, IL-12 p70, and IL-21) studied, only IL-1 β and TNF-α were in the readable ranges. No significant differences were detected. (Figure 5A and 5B).

Accepted manuscript

Other cell populations

Other than the monocytes and T cell populations, we did not find differences in B cells, NK cells, or dendritic cells between the migraineurs and the control group.

ACCEPTED MANUSCRIPT

DISCUSSION

Using peripheral whole blood staining methodology, we found that migraineurs had both myeloid and lymphoid leukocyte surface marker changes compared with the matched control group, which supports the neurogenic inflammatory and autoimmune nature of migraine.

We demonstrated that peripheral blood non-classical monocytes ($CD14^{+}CD16^{++}$) in migraine patients were lower than controls. In migraine, triggering factors such as chronic stress, certain foods, hormonal fluctuation, depression and other unknown causes can generate sterile inflammation in CNS and activation of nociceptors [25]. In the presence of these inflammatory stimuli, circulating inflammatory monocytes can quickly migrate into affected tissues, where they differentiate into macrophages and dendritic cell subsets [26-28]. The patrolling monocytes localize to the endothelium of vascular inflammatory tissues and release inflammatory mediators [26, 29]. Patrolling $CD16^{+}$ monocytes exhibit long-range crawling over the endothelium of vascular tissues [17, 18], and may contribute to the pathogenesis of a wide range of chronic inflammatory diseases, such as obesity, diabetes, atherosclerosis, Chronic Obstructive Pulmonary Disease lung fibrosis, lung cancer, and Alzheimer’s disease [30-32]. This population of monocytes can be detected in perivascular circulation; studies have demonstrated decreased levels of nonclassical monocytes in the peripheral blood, as reported in severe forms of lupus nephritis and MS [15, 33].

Accepted manuscript

ACCEPTED MANUSCRIPT

Current migraine research has provided some evidence of transmigration of non-classical monocytes to the CNS. Nagata et al. searched biomarkers for migraines using microarray analysis in 2009 [11]. In their report, the expression of 15 genes were altered in migraineurs compared to controls, and one-fifth of these genes were associated with cytoskeleton proteins. It has been reported that human non-classical monocytes exhibit crawling behavior on the endothelium both *in vivo* and *in vitro* [16-18, 26, 30]. Non-classical monocytes highly expressed genes associated with cytoskeleton mobility, including Rho GTPase, RHOC and RHOF, and some upstream Rho activators and downstream effectors [16]. These findings are consistent with the Waschbisch clinical study regarding $CD16^{+}$ monocytes migrating to the CNS in MS [15]. Tension-type headache and migraine without aura are among the most common primary headaches in MS patients [34]. $CD16^{+}$ monocytes play a pivotal role in immune-surveillance of the CNS, and they could migrate to the site of inflammation and contribute to the dysfunction of the BBB and CNS autoimmune disease [15] or NeuroAIDS [35]. Both the monocyte chemokine receptor CXCR7 [35, 36] and the integrity of the BBB play roles in inflammatory migraine pathophysiology [37, 38]. Due to limitations imposed by the parent RCT, and the small sample size of this affiliated pilot study, we have not investigated the profile of cerebrospinal fluid (CSF) samples. Immune cell profiling in CSF in future studies could provide additional insights into migraine pathogenesis.

Accepted manuscript

Our study also indicated the potential association between migraine and failure of immunoregulation. We detected a significantly lower peripheral blood CD4+ and its subgroup CD4+CD25+ T cells in the migraine group compared to the matched healthy controls even after controlling for covariates (See Figure 2 and 3). The finding of relatively lower CD4+CD25+ T cells in migraineurs is consistent with a previous migraine study [13]. The difference between ours and Arumugam's study are in the T cells subsets CD4 and CD8 cells. In our migraine group, CD4+ T cells were significantly lower and CD8+ T cells were slightly higher in the migraine group compared with the control group (Figure 3A). However, the differences in CD4+/CD8 ratio was small. (Figure 3B) and appears to be largely due to lower ratio levels in the chronic migraine group (Table 4). These results suggest that failure of immunoregulation could play a role in migraine pathophysiology. In an experimental mouse model of autoimmune encephalomyelitis, non-classical monocytes were shown to be potent suppressors of T cells [39]. Loss of quantity and/or suppressive function of non-classic monocytes may contribute to the development of autoimmune disease. Interestingly, a recent in vitro experiment showed that the CD16+ monocytes, not CD16- classical monocyte, promoted CD4+ T cell trafficking via the endothelial barrier [15]. Provided the CD16+ monocytes did migrate to the CNS, they then may enhance T cell entry into the CNS, the latter further facilitating CD16+ monocytes' attachment to the microvasculature and contributing to

Accepted manuscript

ACCEPTED MANUSCRIPT

the breakdown of the BBB. This could explain why peripheral blood CD4+ T cell numbers were lower in our study.

Considering that about half of the recruited patients also fall into chronic migraine or suffer from overuse of any pain medication, we did further analysis based on these two categories themselves. The results are in supplemental table 1. We found that chronic migraine patients demonstrated the most significant changes compared with their matched volunteers, including non-classical monocytes, CD4+ T cells, CD4/CD8 ratio, CD18 MFI on CD4+ and CD8+ T cells, CD49 MFI on CD4+ T cells, CD36, and CD4+CD25+ T cell populations. We realized that the sample size was even smaller, and the pathophysiology of the transformation from episodic to chronic migraine can not be concluded at this time.

Accepted manuscript

CD36 is a transmembrane protein expressed in various types of cells, including adipocytes, monocytes, macrophages, platelets, endothelial, and muscle cells [40]. This protein belongs to the scavenger receptor [40, 41] and binds to lipoproteins, apoptotic cells, and long-chain fatty acids; thus, CD36 is also known as fatty acid translocase [42]. Coburn et al. demonstrated that long chain fatty acid uptake and utilization have been defective in CD36 knockout mice [43]. Our team reported that a dietary intervention with increased omega-3 fatty acids helped to reduce headache pain [44, 45], and it has been known that omega-3 could induce the CD36 mRNA expression in an animal model [46, 47]. CD36 can bind to three

classes of ligands including modified phospholipids, long chain fatty acids and domains of thrombospondin homologs [40]. Broad expression of CD36 on different cells suggests this multi-functional receptor could be involved in a broad spectrum of diseases [40, 48-50]. The effects of high dietary omega-3 and low omega-6 targeted alteration to treat migraine in the parent randomized clinical trial suggested that the binding of long chain fatty acid to CD36 might play vital roles in chronic headache [44] and possibly for migraine [45]. Indeed, predominant monocytes and platelets gene up-regulations are characteristic for migraine [10]. CD36 binding to domains of thrombospondin homologs and the downstream inflammation activation might play a role in migraine pathophysiology, but the details are still elusive.

Accepted manuscript

T-cell subset and expression of integrins are reduced to potentially facilitate the lymphocyte transmigration to the CNS. LFA-1 is expressed on white blood cells including lymphocytes and other leukocytes and plays a crucial role in the emigration of leukocytes, leaving the bloodstream to enter the tissues. It is a heterodimeric glycoprotein with non-covalently linked subunits alpha (CD11a) and beta (CD18). Empl et al.’s study reported that migraine-with-aura patients had significantly lower LFA-1 expression on both CD4+ and CD8+ T cells than that of controls [51]. Sarchielli and colleagues found that LFA -1 on CD4+ and CD8+ T cells were progressively down-regulated at two and four hours after migraine attack onset [52].

ACCEPTED MANUSCRIPT

Previous research has reported that elevated levels of biomarkers of inflammation are linked to migraine [5, 53, 54]. In the early phase of inflammation, the release of excessive amounts of pro-inflammatory cytokines and lipid-mediators is tied to the pathogenesis of organ dysfunction. Cytokines and chemokines are essential molecular and pain mediators in neurovascular inflammation. Some proinflammatory cytokines levels increased after migraine attacks [25-27]. In addition, cytokines might play a critical role in the initiation and persistence of pain by activating nociceptive sensory neurons [55, 56]. The most relevant cytokines related to migraines include IL-1, IL-2, IL-4, IL-6, IL-10, TNF-α, and TGF-β [9, 57-59]. Recent studies investigating neuroinflammation in migraine reveal the role of inflammasome via inflammasome complex players including IL-1β and IL-18 [60]. In this study, we did not find significant changes of inflammatory cytokines from serum (data not provided) or *ex vivo* LPS stimulated PBMCs between migraine patients and matched control groups. Although IL-6 has been reported with higher production from this population in the Koon's study [61], we found a reduced non-classical monocyte population in the peripheral blood, and this is likely to be a reason for not finding significantly increased IL-6 production. Moreover, condition of *ex vivo* LPS stimulation needs to be investigated in the future.

Accepted manuscript

There are some limitations in this pilot study. For instance, the sample size was relatively small, and the *ex vivo* LPS stimulation of PBMC for cytokine production used only a single 24-hour stimulation time point. Because of

ACCEPTED MANUSCRIPT

limited patient samples available from the clinical parent study, CSF samples were not examined.

In summary, we observed that the percentage of peripheral non-classical monocyte ($CD14^{+}CD16^{++}$) in migraine is lower than in controls, suggesting possible migration of the $CD14^{+}CD16^{++}$ population into the endothelium of cranial vessels. This migration potentially plays a role in releasing inflammatory mediators, leading to migraine pathogenesis. Future research regarding the levels of non-classic monocyte population in the CNS (for example, in cerebrospinal fluid) would be of interest to confirm the potential migration of the $CD16^{+}$ monocyte population. Migraine appears to be an inflammatory disease with a lowered $CD4^{+}$T cell population, specifically a lower $CD4^{+}CD25^{+}$ T cell population. These preliminary findings need to be confirmed with studies conducted on a larger sample size of patients with migraine.

If future studies can directly confirm the migration of the non-classical monocytes in the CNS of migraine patients, potential therapeutic strategies may be broadened. For example, since $CD16^{+}$ monocytes express CXCR7 on cell surface; using CXCR7 antagonist might help reduce the non-classical monocytes transmigration across the BBB [35]. This study also provides additional evidence for the use of targeted alterations in dietary linolenic acid and n-3 $EPA^{+}DHA$ in chronic migraineurs [44, 45]. Neuroinflammation pathways, specifically those involving inflammasome proteins, such as IL-1β,

Accepted manuscript

ACCEPTED MANUSCRIPT

IL-18, and caspase-1, seem promising candidates as biomarkers or treatment targets in migraine [60], providing some interesting direction for further study.

Conclusions:

Our results suggest that migraine is associated with dysregulated peripheral immune homeostasis and that neuroinflammation and autoimmune may play a role in its pathophysiology.

Accepted manuscript

Abbreviations:

BBB: blood brain barrier

BMI: body mass index

CNS: central nervous system

LFA-1: Lymphocyte function-associated antigen 1

MFI: mean fluorescence intensity

MS: multiple sclerosis

PBMC: peripheral blood mononuclear cells

PMNs: Polymorphonuclear leukocytes

VLA-4: very late antigen-4

n-3: Omega-3

ACCEPTED MANUSCRIPT

DECLARATIONS

Protocols and ethics approval and consent to participate

This study was carried out according to the guidelines and regulations for human research ethics and good clinical and lab practice. The Institutional Review Board of the University of North Carolina at Chapel Hill reviewed and approved the experimental protocols (Study # 15-1139). Participants in the study gave informed consent for their participation, including completing questionnaires and providing blood specimens for analysis.

Accepted manuscript

Consent for publication

Not applicable.

Availability of data and materials

The datasets used and /or analysed during the current study are not able to share due to ethnical/legal restrictions. UNC Health/CDWH only permits data use on a project-by-project basis (i.e., a given IRB) and any disclosures outside of UNC require a data use agreement.

Competing interests

The authors declare no conflict of interest. The contents of this publication are solely the authors' responsibility and so do not represent the official views and opinions of the N.I.H.

Funding

ACCEPTED MANUSCRIPT

The parent grant funded by NIH NCCIH R01-AT007813 (2013-2018) was awarded to Dr. John D. Mann as principal investigator at the UNC School of Medicine, Chapel Hill, NC. The study's first author (H.L.) was financially supported by NIH T32 Fellowship NCCIH 5T32AT003378 and the study itself was supported by an NC TraCS2K from UNC Translational and Clinical Science Institute award 2KR691507 (H.L.).

Accepted manuscript

Authors' contributions

H.L. designed the experiments, performed the experiments, and was the primary author of this manuscript. Q.F. contributed to experimental design, data processing, and statistical analysis. K.P. recruited and consented the control group, performed experiments with H.L., and critically revised this manuscript. Y.S. processed the flow cytometry raw data, statistical analysis, and generated related graphs. K.R.F. was a Co-I on the parent R01, and was involved with the NC TraCS 2k grant application (protocol), data analysis and editing the manuscript as the senior author. S.G. and J.D.M. participated in editing the manuscript and management of the NIH T-32 fellowship grant NCCIH 5T32AT003378, as well as participating as Co-I and PI on the parent RO1.

Acknowledgments

The postdoctoral office location was at the Program on Integrative Medicine, Department of Physical Medicine and Rehabilitation, UNC School of Medicine, Chapel Hill, NC. The benchwork was conducted in Dr. Lishan Su's laboratory at the Department of Microbiology and Immunology, School of Medicine, UNC-Chapel Hill. Dr. Guangming Li offered generous technical support. Dr.

ACCEPTED MANUSCRIPT

Can Cui helped set up the strategy for the FlowJo analysis. Drs. Xuewei Zhu, Christopher Ramsden, Lishan Su, and Peng Liu generously helped proofread the manuscript. Ms. Theresa Watson from St. Mary-of-the-Woods College and Mr. Steve Baker helped with figure editing.

Accepted manuscript

ACCEPTED MANUSCRIPT

REFERENCES

[]

1. Stovner LJ, Hagen K, Jensen R, Katsarava Z, Lipton RB, Scher AI, et al. The global burden of headache: a documentation of headache prevalence and disability worldwide. Cephalalgia. 2007;27 3:193-210; doi: 10.1111/j.1468-2982.2007.01288.x.
2. Mann JD, Faurot KR, MacIntosh B, Palsson OS, Suchindran CM, Gaylord SA, et al. A sixteen-week three-armed, randomized, controlled trial investigating clinical and biochemical effects of targeted alterations in dietary linoleic acid and n-3 EPA+DHA in adults with episodic migraine: Study protocol. Prostaglandins Leukot Essent Fatty Acids. 2018;128:41-52; doi: 10.1016/j.plefa.2017.11.002.
3. Williamson DJ, Hargreaves RJ. Neurogenic inflammation in the context of migraine. Microsc Res Tech. 2001;53 3:167-78; doi: 10.1002/jemt.1081.
4. Waeber C, Moskowitz MA. Migraine as an inflammatory disorder. Neurology. 2005;64 10 suppl 2:S9-S15; doi: 10.1212/WNL.64.10_suppl_2.S9.
5. Tietjen GE, Khubchandani J, Herial N, Palm-Meinders IH, Koppen H, Terwindt GM, et al. Migraine and vascular disease biomarkers: A population-based case-control study. Cephalalgia. 2017:333102417698936; doi: 10.1177/0333102417698936.
6. Mahajan R, Mahajan R, Juneja A, Anand K. Inflammatory biomarkers in migraine- A prospective observational study. International Journal of Scientific Research. 2020.
7. Sarıcam G. Relationship between migraine headache and hematological parameters. Acta neurologica Belgica 2020.
8. Kemper RH, Meijler WJ, Korf J, Ter Horst GJ. Migraine and function of the immune system: a meta-analysis of clinical literature published between 1966 and 1999. Cephalalgia. 2001;21 5:549-57; doi: 10.1046/j.1468-2982.2001.00196.x.
9. Bruno PP, Carpino F, Carpino G, Zicari A. An overview on immune system and migraine. Eur Rev Med Pharmacol Sci. 2007;11 4:245-8.
10. Du X, Tang Y, Xu H, Lit L, Walker W, Ashwood P, et al. Genomic profiles for human peripheral blood T cells, B cells, natural killer cells, monocytes, and polymorphonuclear cells: Comparisons to ischemic stroke, migraine, and Tourette syndrome. Genomics. 2006;87 6:693-703; doi: https://doi.org/10.1016/j.ygeno.2006.02.003.
11. Nagata E, Hattori H, Kato M, Ogasawara S, Suzuki S, Shibata M, et al. Identification of biomarkers associated with migraine with aura. Neurosci Res. 2009;64 1:104-10; doi: 10.1016/j.neures.2009.02.001.
12. Gerring ZF, Powell JE, Montgomery GW, Nyholt DR. Genome-wide analysis of blood gene expression in migraine implicates immune-inflammatory pathways. Cephalalgia. 2017:333102416686769; doi: 10.1177/0333102416686769.
13. Arumugam M, Parthasarathy V. Reduction of CD4+CD25+ regulatory T-cells in migraine: Is migraine an autoimmune disorder? J Neuroimmunol. 2016;290:54-9; doi: http://dx.doi.org/10.1016/j.jneuroim.2015.11.015.

Accepted manuscript

ACCEPTED MANUSCRIPT

14. Sampath P, Moideen K, Ranganathan UD, Bethunaickan R. Monocyte Subsets: Phenotypes and Function in Tuberculosis Infection. Frontiers in Immunology. 2018;9 1726; doi: 10.3389/fimmu.2018.01726.
15. Waschbisch A, Schroder S, Schraudner D, Sammet L, Weksler B, Melms A, et al. Pivotal Role for CD16+ Monocytes in Immune Surveillance of the Central Nervous System. J Immunol. 2016;196 4:1558-67; doi: 10.4049/jimmunol.1501960.
16. Wong KL, Yeap WH, Tai JJY, Ong SM, Dang TM, Wong SC. The three human monocyte subsets: implications for health and disease. Immunol Res. 2012;53 1:41-57; doi: 10.1007/s12026-012-8297-3.
17. Auffray C, Sieweke MH, Geissmann F. Blood Monocytes: Development, Heterogeneity, and Relationship with Dendritic Cells. Annu Rev Immunol. 2009;27 1:669-92; doi: doi:10.1146/annurev.immunol.021908.132557.
18. Saha P, Geissmann F. Toward a functional characterization of blood monocytes. Immunol Cell Biol. 2011;89 1:2-4.
19. Mukherjee R, Kanti Barman P, Kumar Thatoi P, Tripathy R, Kumar Das B, Ravindran B. Non-Classical monocytes display inflammatory features: Validation in Sepsis and Systemic Lupus Erythematous. Scientific Reports. 2015;5 1:13886; doi: 10.1038/srep13886.
20. Veenstra M, León-Rivera R, Li M, Gama L, Clements JE, Berman JW. Mechanisms of CNS Viral Seeding by HIV+ CD14+ CD16+ Monocytes: Establishment and Reseeding of Viral Reservoirs Contributing to HIV-Associated Neurocognitive Disorders. mBio. 2017;8 5:e01280-17; doi: 10.1128/mBio.01280-17.
21. van Sleen Y, Wang Q, van der Geest KSM, Westra J, Abdulahad WH, Heeringa P, et al. Involvement of Monocyte Subsets in the Immunopathology of Giant Cell Arteritis. Scientific reports. 2017;7 1:6553-; doi: 10.1038/s41598-017-06826-4.
22. Marski M, Ye AL, Abraham C. CD18 is required for intestinal T cell responses at multiple immune checkpoints. J Immunol. 2007;178 4:2104-12; doi: 10.4049/jimmunol.178.4.2104.
23. Nicholson JK, Rao PE, Calvelli T, Stetler-Stevenson M, Browning SW, Yeung L, et al. Artifactual staining of monoclonal antibodies in two-color combinations is due to an immunoglobulin in the serum and plasma. Cytometry. 1994;18 3:140-6; doi: 10.1002/cyto.990180305.
24. Buse DC, Reed ML, Fanning KM, Bostic R, Dodick DW, Schwedt TJ, et al. Comorbid and co-occurring conditions in migraine and associated risk of increasing headache pain intensity and headache frequency: results of the migraine in America symptoms and treatment (MAST) study. The Journal of Headache and Pain. 2020;21 1:23; doi: 10.1186/s10194-020-1084-y.
25. Ramachandran R. Neurogenic inflammation and its role in migraine. Seminars in Immunopathology. 2018;40 3:301-14; doi: 10.1007/s00281-018-0676-y.
26. Thomas G, Tacke R, Hedrick CC, Hanna RN. Nonclassical patrolling monocyte function in the vasculature. Arterioscler Thromb Vac Biol. 2015;35 6:1306-16; doi: 10.1161/atvbaha.114.304650.
27. Italiani P, Boraschi D. From Monocytes to M1/M2 Macrophages: Phenotypical vs. Functional Differentiation. Frontiers in Immunology. 2014;5:514; doi: 10.3389/fimmu.2014.00514.
28. Martinez FO, Gordon S. The M1 and M2 paradigm of macrophage activation: time for reassessment. F1000Prime Reports. 2014;6:13; doi: 10.12703/P6-13.

Accepted manuscript

ACCEPTED MANUSCRIPT

29. Abdulkhaleq LA, Assi MA, Abdullah R, Zamri-Saad M, Taufiq-Yap YH, Hezmee MNM. The crucial roles of inflammatory mediators in inflammation: A review. Veterinary world. 2018;11 5:627-35; doi: 10.14202/vetworld.2018.627-635.
30. Kapellos TS, Bonaguro L, Gemünd I, Reusch N, Saglam A, Hinkley ER, et al. Human Monocyte Subsets and Phenotypes in Major Chronic Inflammatory Diseases. Frontiers in immunology. 2019;10:2035-; doi: 10.3389/fimmu.2019.02035.
31. Min D, Brooks B, Wong J, Salomon R, Bao W, Harrisberg B, et al. Alterations in Monocyte CD16 in Association with Diabetes Complications. Mediators Inflamm. 2012;2012:649083; doi: 10.1155/2012/649083.
32. Narasimhan PB, Marcovecchio P, Hamers AAJ, Hedrick CC. Nonclassical Monocytes in Health and Disease. Annu Rev Immunol. 2019;37 1:439-56; doi: 10.1146/annurev-immunol-042617-053119.
33. Barrera García A, Gómez-Puerta JA, Arias LF, Burbano C, Restrepo M, Vanegas AL, et al. Infiltrating CD16+ Are Associated with a Reduction in Peripheral CD14+CD16++ Monocytes and Severe Forms of Lupus Nephritis. Autoimmune Diseases. 2016;2016:9324315; doi: 10.1155/2016/9324315.
34. La Mantia L, Prone V. Headache in multiple sclerosis and autoimmune disorders. Neurol Sci. 2015;36 Suppl 1:75-8; doi: 10.1007/s10072-015-2146-9.
35. Veenstra M, Williams DW, Calderon TM, Anastos K, Morgello S, Berman JW. Frontline Science: CXCR7 mediates CD14+CD16+ monocyte transmigration across the blood brain barrier: a potential therapeutic target for NeuroAIDS. J Leukoc Biol. 2017;102 5:1173-85; doi: 10.1189/jlb.3HI0517-167R.
36. Chatterjee M, von Ungern-Sternberg SNI, Seizer P, Schlegel F, Büttcher M, Sindhu NA, et al. Platelet-derived CXCL12 regulates monocyte function, survival, differentiation into macrophages and foam cells through differential involvement of CXCR4–CXCR7. Cell Death & Disease. 2015;6 11:e1989-e; doi: 10.1038/cddis.2015.233.
37. Goadsby PJ, Holland PR, Martins-Oliveira M, Hoffmann J, Schankin C, Akerman S. Pathophysiology of Migraine: A Disorder of Sensory Processing. Physiol Rev. 2017;97 2:553-622; doi: 10.1152/physrev.00034.2015.
38. DosSantos MF, Holanda-Afonso RC, Lima RL, DaSilva AF, Moura-Neto V. The role of the blood-brain barrier in the development and treatment of migraine and other pain disorders. Front Cell Neurosci. 2014;8:302; doi: 10.3389/fncel.2014.00302.
39. Slaney CY, Toker A, La Flamme A, Bäckström BT, Harper JL. Naïve blood monocytes suppress T-cell function. A possible mechanism for protection from autoimmunity. Immunol Cell Biol. 2011;89 1:7-13; doi: 10.1038/icb.2010.110.
40. Choromańska B, Myśliwiec P, Choromańska K, Dadan J, Chabowski A. The role of CD36 receptor in the pathogenesis of atherosclerosis. Adv Clin Exp Med. 2017;26 4:717-22; doi: 10.17219/acem/62325.
41. Xie S, Lee YF, Kim E, Chen LM, Ni J, Fang LY, et al. TR4 nuclear receptor functions as a fatty acid sensor to modulate CD36 expression and foam cell formation. Proc Natl Acad Sci U S A. 2009;106 32:13353-8; doi: 10.1073/pnas.0905724106.
42. Zheng JS, Chen J, Wang L, Yang H, Fang L, Yu Y, et al. Replication of a Gene-Diet Interaction at CD36, NOS3 and PPARG in Response to Omega-3 Fatty

Accepted manuscript

ACCEPTED MANUSCRIPT

Acid Supplements on Blood Lipids: A Double-Blind Randomized Controlled Trial. EBioMedicine. 2018;31:150-6; doi: 10.1016/j.ebiom.2018.04.012.
43. Coburn CT, Knapp FF, Jr., Febbraio M, Beets AL, Silverstein RL, Abumrad NA. Defective uptake and utilization of long chain fatty acids in muscle and adipose tissues of CD36 knockout mice. J Biol Chem. 2000;275 42:32523-9; doi: 10.1074/jbc.M003826200.
44. Ramsden CE, Faurot KR, Zamora D, Suchindran CM, Macintosh BA, Gaylord S, et al. Targeted alteration of dietary n-3 and n-6 fatty acids for the treatment of chronic headaches: a randomized trial. Pain. 2013;154 11:2441-51; doi: 10.1016/j.pain.2013.07.028.
45. Ramsden CE, Zamora D, Faurot KR, MacIntosh B, Horowitz M, Keyes GS, et al. Dietary alteration of n-3 and n-6 fatty acids for headache reduction in adults with migraine: randomized controlled trial. BMJ. 2021;374:n1448; doi: 10.1136/bmj.n1448.
46. Alexander Aguilera A, Hernández Díaz G, Lara Barcelata M, Angulo Guerrero O, Oliart Ros RM. Induction of Cd36 expression elicited by fish oil PUFA in spontaneously hypertensive rats. The Journal of Nutritional Biochemistry. 2006;17 11:760-5; doi: https://doi.org/10.1016/j.jnutbio.2005.12.007.
47. Yuan F, Wang H, Tian Y, Li Q, He L, Li N, et al. Fish oil alleviated high-fat diet-induced non-alcoholic fatty liver disease via regulating hepatic lipids metabolism and metaflammation: a transcriptomic study. Lipids Health Dis. 2016;15:20; doi: 10.1186/s12944-016-0190-y.
48. Febbraio M, Silverstein RL. CD36: Implications in Cardiovascular Disease. The international journal of biochemistry & cell biology. 2007;39 11:2012-30; doi: 10.1016/j.biocel.2007.03.012.
49. Kennedy DJ, Kuchibhotla S, Westfall KM, Silverstein RL, Morton RE, Febbraio M. A CD36-dependent pathway enhances macrophage and adipose tissue inflammation and impairs insulin signalling. Cardiovasc Res. 2011;89 3:604-13.
50. Sharif O, Matt U, Saluzzo S, Lakovits K, Haslinger I, Furtner T, et al. The scavenger receptor CD36 downmodulates the early inflammatory response while enhancing bacterial phagocytosis during pneumococcal pneumonia. J Immunol. 2013;190 11:5640-8; doi: 10.4049/jimmunol.1202270.
51. Empl M, Sostak P, Breckner M, Riedel M, Muller N, Gruber R, et al. T-cell subsets and expression of integrins in peripheral blood of patients with migraine. Cephalalgia. 1999;19 8:713-7; discussion 697; doi: 10.1046/j.1468-2982.1999.019008713.x.
52. Sarchielli P, Alberti A, Baldi A, Coppola F, Rossi C, Pierguidi L, et al. Proinflammatory Cytokines, Adhesion Molecules, and Lymphocyte Integrin Expression in the Internal Jugular Blood of Migraine Patients Without Aura Assessed Ictally. Headache: The Journal of Head and Face Pain. 2006;46 2:200-7; doi: 10.1111/j.1526-4610.2006.00337.x.
53. Gormley P, Winsvold BS, Nyholt DR, Kallela M, Chasman DI, Palotie A. Migraine genetics: from genome-wide association studies to translational insights. Genome Med. 2016;8 1:86; doi: 10.1186/s13073-016-0346-4.
54. Khaiboullina SF, Mendelevich EG, Shigapova LH, Shagimardanova E, Gazizova G, Nikitin A, et al. Cerebellar Atrophy and Changes in Cytokines Associated with the CACNA1A R583Q Mutation in a Russian Familial Hemiplegic Migraine Type 1 Family. Front Cell Neurosci. 2017;11:263; doi: 10.3389/fncel.2017.00263.

Accepted manuscript

55. Zhang J-M, An J. Cytokines, inflammation, and pain. Int Anesthesiol Clin. 2007;45 2:27-37; doi: 10.1097/AIA.0b013e318034194e.
56. Patti F, Nicoletti A, Pappalardo A, Castiglione A, Lo Fermo S, Messina S, et al. Frequency and severity of headache is worsened by Interferon-β therapy in patients with multiple sclerosis. Acta Neurol Scand. 2012;125 2:91-5; doi: 10.1111/j.1600-0404.2011.01532.x.
57. Franceschini A, Vilotti S, Ferrari MD, van den Maagdenberg AMJM, Nistri A, Fabbretti E. TNFα levels and macrophages expression reflect an inflammatory potential of trigeminal ganglia in a mouse model of familial hemiplegic migraine. PloS one. 2013;8 1:e52394-e; doi: 10.1371/journal.pone.0052394.
58. Martelletti P, Zicari A, Realacci M, Fiore G, De Filippis S, Stirparo G, et al. Expression of NOS–2, COX–2 and Th1/Th2 cytokines in migraine. The Journal of Headache and Pain. 2001;2 Suppl 1:s51-s6; doi: 10.1007/s101940170010.
59. Munno I, Centonze V, Marinaro M, Bassi A, Lacedra G, Causarano V, et al. Cytokines and Migraine: Increase of IL-5 and IL-4 Plasma Levels. Headache: The Journal of Head and Face Pain. 1998;38 6:465-7; doi: 10.1046/j.1526-4610.1998.3806465.x.
60. Kursun O, Yemisci M, van den Maagdenberg AMJM, Karatas H. Migraine and neuroinflammation: the inflammasome perspective. The Journal of Headache and Pain. 2021;22 1:55; doi: 10.1186/s10194-021-01271-1.
61. Kong BS, Kim Y, Kim GY, Hyun J-W, Kim S-H, Jeong A, et al. Increased frequency of IL-6-producing non-classical monocytes in neuromyelitis optica spectrum disorder. Journal of Neuroinflammation. 2017;14 1:191; doi: 10.1186/s12974-017-0961-z.

Accepted manuscript

ACCEPTED MANUSCRIPT

A

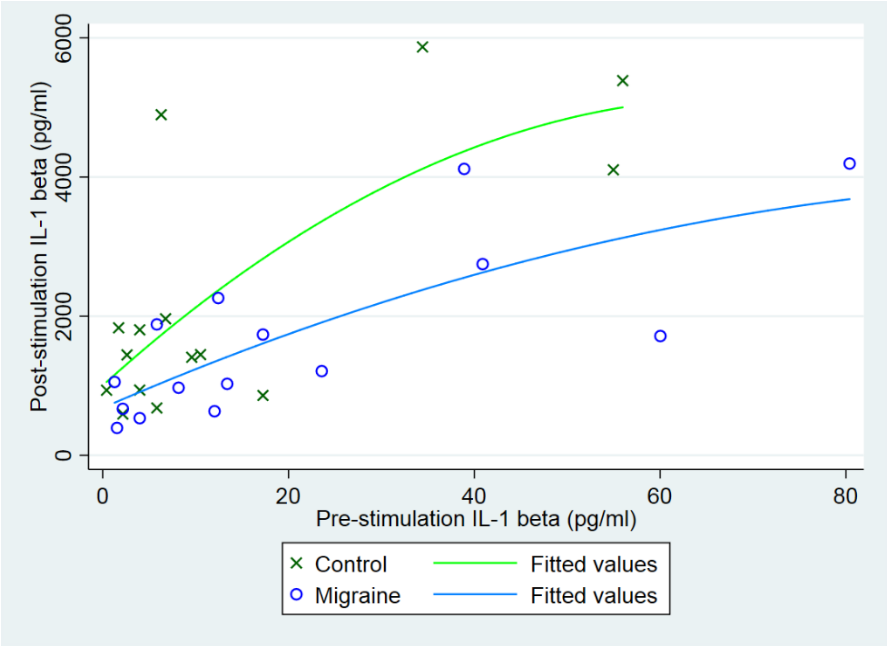

B

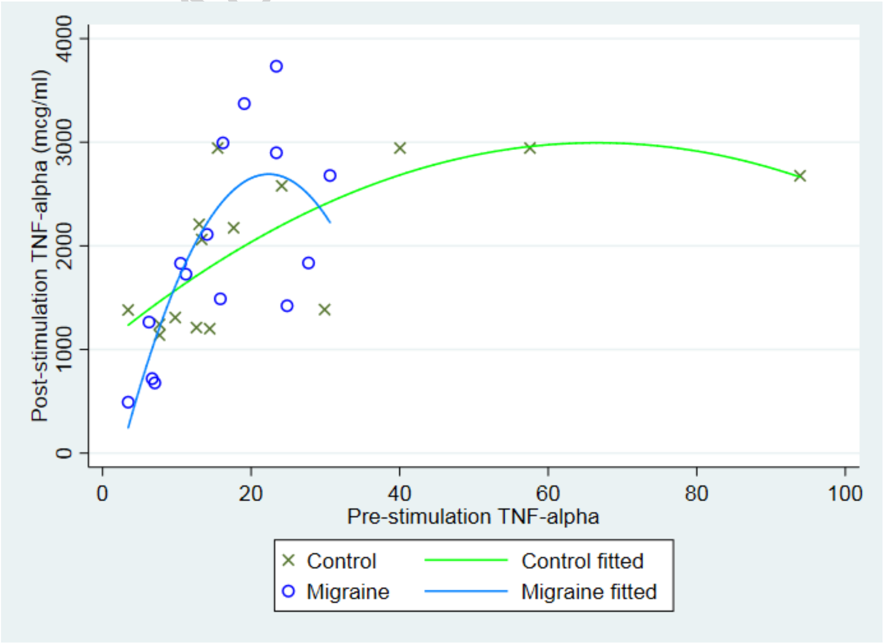

Figure 5. Cytokine analysis with 24-hour LPS stimulation revealed no significant difference in inflammatory cytokine levels (IL-1 beta and TNF-a) between migraineurs and controls. Pre-stimulation and post-stimulation of IL-1β (Figure 5A) and TNF-α (Figure 5B) were processed using Stata software, no significant changes were found.

ACCEPTED MANUSCRIPT

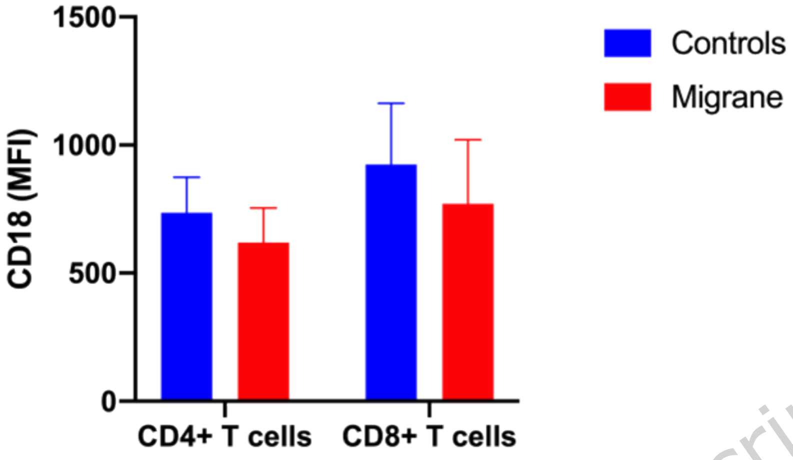

Figure 4. Both CD4+ helper T cells and CD8+ killer T cells demonstrated lowered expression by mean fluorescence intensity (MFI) of integrin CD18, but not significantly. MFI of CD18 was lower in both CD4+ T cells and CD8+ T cells in migraineurs compared with controls, with $p=0.06$ and $p=0.09$, respectively. Data are represented as means ± SD, analysis was determined by nonparametric Wilcoxon signed rank test.

Accepted manuscript

ACCEPTED MANUSCRIPT

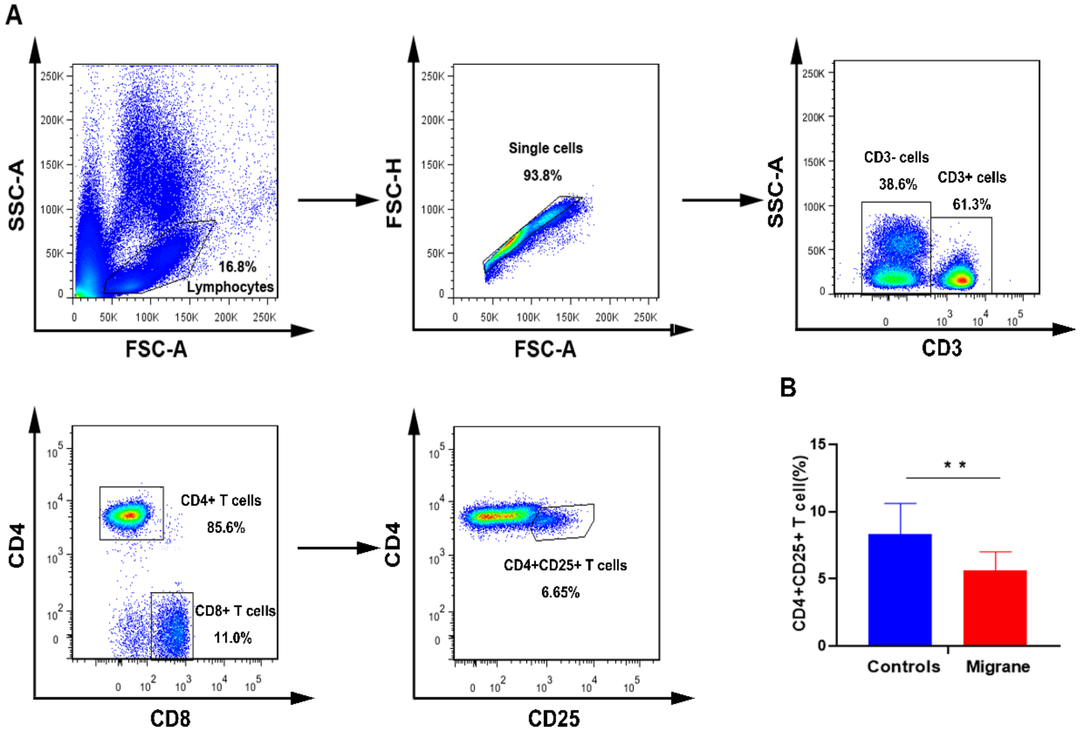

Figure 3. CD4+CD25+ T cell populations from migraineurs were significantly lower than controls. (A) The lymphocytes and monocytes populations are highlighted, and the single cell population was selected only. CD3+ and CD4+ population were selected, respectively. Finally, CD4+CD25+ population was highlighted. (B) CD4+CD25+ T cell populations from migraineurs were significantly lower than controls (5.66±1.35 vs. 8.29±2.31), **P=0.001. Data are represented as means ± SD, significance was determined by nonparametric Wilcoxon signed rank test.

ACCEPTED MANUSCRIPT

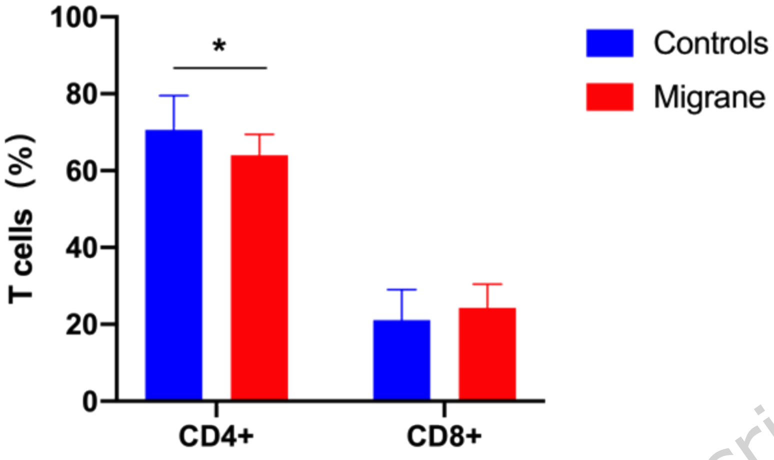

Figure 2. CD4 T cell populations from migraineurs were significantly lower than controls. (A) CD4 T cell populations from migraineurs were significantly lower than controls (64.0±5.45 vs. 70.6±8.92), *p=0.035. (B) CD4/CD8 ratio was lowed in the migraineurs, yet the change was not significant (not shown). *p<0.05, **p<0.01. Data are represented as means ± SD, significance was determined by nonparametric Wilcoxon signed rank test.

Accepted manuscript

ACCEPTED MANUSCRIPT

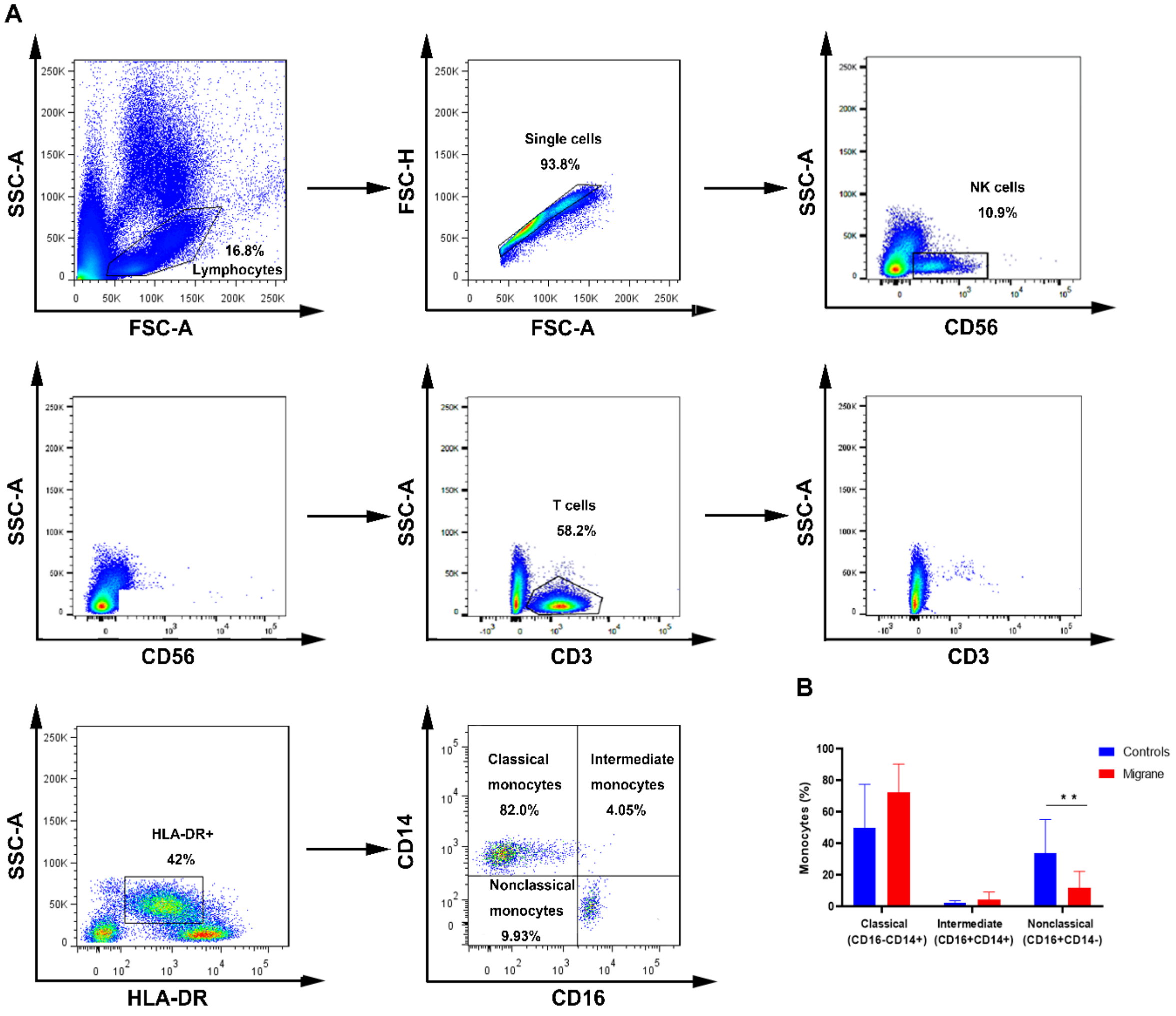

Figure 1. Non-classical monocytes (CD14+CD16++) were significantly lower in the peripheral blood of migraine group compared with the matched control group. (A) First, the lymphocytes and monocytes populations are highlighted, and the single cell population was selected only. Then NK cells, T cells were removed. HLA-DR and SSC-A subset was selected, and three populations within the monocytes were identified by CD14 and CD16 markers. (B) Bar graph showed that non-classical monocytes (CD14+CD16++) were significantly lower (11.9 ±10.5 vs. 33.9 ±21.4), **p=0.005 in the peripheral blood of migraine group compared with the matched control group, *p<0.05, **p<0.01. Data are represented as means ± SD, significance was determined by nonparametric Wilcoxon signed rank test.

ACCEPTED MANUSCRIPT

Table 4. Means, standard deviations, and medians for groups defined by control and migraine status

Variable	Controls		Migraine		
	n=15		n=15		
	Mean (SD)	Median	Mean (SD)	Median	p value*
Monocytes (%)					
Classical (CD16-CD14+)	49.9 (27.6)	48.7	72.3 (18.1)	78.5	0.06
Intermediate (CD16+CD14+)	2.23 (1.51)	1.86	4.44 (4.88)	3.44	0.09
Nonclassical (CD16+CD14-)	33.9 (21.4)	33.2	11.9 (10.5)	9.16	0.005
T cells (%)					
CD4+	70.6 (8.92)	66.3	64.0 (5.45)	63.3	0.035
CD8+	21.1 (7.92)	22.3	24.3 (6.16)	22.9	0.3
CD4/CD8	4.02 (2.10)	3.23	2.80 (0.75)	2.83	0.09
CD18(MFI) CD4	735 (139)	730	619 (135)	608	0.06
CD18(MFI) CD8	924 (239)	938	771 (250)	715	0.09
CD49(MFI) CD4	1247 (240)	1275	1195 (341)	1047	0.9
CD49(MFI) CD8	1468 (321)	1424	1327 (332)	1358	0.4
CD36	352 (255)	242	272 (191)	224	0.4
CD4+CD25+	8.29 (2.31)	7.69	5.66 (1.35)	5.08	0.001

*p values in this table are based on comparisons with the controls based on the nonparametric Wilcoxon signed rank test

Accepted manuscript

ACCEPTED MANUSCRIPT

Table 3. Migraine, headache (HA), pain information and overuse of pain medication

Study ID	Number of HA per 30 days	Number of migraines per 30 days	Comorbid pain	Aura	Meets chronic migraine criteria	Percent of days with HA (A)	Percent of days with Migraine (B)	Percent of headaches that are migraines (A/B)	Meets criteria for overuse of any pain medication	Triptan overuse	NSAID /ASA overuse	Overuse of opioids	Overuse multiple drug classes
M1133	9	9	TMJ	1	0	31%	29%	91%	**0**	0	0	0	0
M1139	26	20	None	1	1	87%	65%	75%	**1**	0	1	0	1
M1140	30	19	IBS	0	1	100%	62%	62%	**0**	0	0	0	0
M1142	30	20	Fibromyalgia	0	1	100%	68%	68%	**1**	0	0	0	1
M1143	30	20	Fibromyalgia	0	1	100%	68%	68%	**0**	0	0	0	0
M1144	17	7	IBS	0	0	57%	22%	38%	**0**	0	0	0	0
M1145	24	9	Back pain	0	1	81%	29%	35%	**1**	0	0	1	1
M1149	10	6	None	0	0	34%	21%	60%	**1**	0	0	0	1
M1152	9	6	None	1	0	29%	20%	70%	**0**	0	0	0	0
M1153	19	12	None	0	1	64%	41%	64%	**1**	0	0	0	1
M1154	17	6	Arthritis	1	0	55%	21%	38%	**1**	0	0	0	1
M1155	16	8	Back pain	0	1	52%	28%	54%	**0**	0	0	0	0
M1156	13	5	None	0	0	43%	18%	42%	**1**	0	0	0	1

Accepted manuscript

ACCEPTED MANUSCRIPT

M1158	12	11	Arthritis	0	0	41%	38%	92%	**1**	0	0	0	1
M1159	16	10	None	1	1	55%	32%	58%	**0**	0	0	0	0
Mean	18.6	11.2	Sum	5	8				8				
SD	7.7	5.7	Percent	33%	53%				53%				

Definition of chronic migraine: Headache occurring on 15 or more days/month for more than 3 months, which, on at least 8 days/month, has the features of migraine headache.

Accepted manuscript

ACCEPTED MANUSCRIPT

Table 2. Demographic comparison between migraine and its matched control group

	Migraine sample	Controls
Male gender*	3 (20%)	3 (20%)
Age*	39.3 (12.2)	39.7 (12.2)
Body Mass Index*	27.2 (4.6)	27.4 (4.8)
Race/ethnicity		
NonHispanic White	12 (80%)	10 (67%)
NonHispanic Black	2 (13%)	2 (13%)
Other**	1 (7%)	3 (20%)
Education		
High school or less	3 (20%)	0 (0%)
Some college	2 (13%)	5 (38%)
Bachelor's degree	5 (33%)	4 (31%)
Advanced degree	5 (33%)	4 (31%)
Income		
$20,000 or less	1 (7%)	0 (0%)
$21,000 - 40,000	1 (7%)	4 (29%)
$41,000 - 60,000	2 (13%)	4 (29%)
$61,000 - 80,000	2 (13%)	3 (21%)
More than $80,000	7 (47%)	3 (21%)
Relationship status		
Living with partner	12 (80%)	7 (50%)
Not living with partner	3 (20%)	7 (50%)
Smoking status		
Current smoker	1 (7%)	0 (0%)
Former smoker	4 (27%)	3 (21%)
Nonsmoker	10 (67%)	11 (79%)
Alcohol intake		
None or rare	13 (87%)	10 (71%)
Occasional	2 (13%)	4 (29%)

Continuous variables presented as mean (standard deviation)

Categorical variables presented as n(%)

* Matching variables

Accepted manuscript

ACCEPTED MANUSCRIPT

Table 1. The Three flow cytometry panels of antibodies with the intention of different population screening. Following BD whole blood staining protocols, we chose three panels of antibodies, with each panel containing eight antibodies as shown in the table.

Monocyte, its subsets and activation markers		NK, T cells, their subsets and activation markers		B, Dendritic cells, their subsets and activation markers	
CD3	APC-H7	CD8	V500	CD3	APC-H7
CD36	PerCP-Cy5.5	CD4	PerCP-Cy5.5	CD4	PerCP-Cy5.5
CD14	V500	CD3	APC-H7	CD11c	BV510
CD16	BV421	CD16	BV421	HLA-DR	BB515
HLD-DR	BB515	CD25	BB515	CD123	BV421
CD56	PE-Cy7	CD18	PE	CD86	APC
CD86	APC	CD56	PE-Cy7	CD19	PE-Cy7
CD163	PE	CD49d	APC	CD80	PE

Accepted manuscript
