# Supplementary material for: Leukocyte inflammatory phenotype and function in migraine patients compared with matched non-migraine volunteers: a pilot study
Source: BMC Neurol. 2022 Jul 27;22:278. doi: 10.1186/s12883-022-02781-4 (PMC9327171; doi:10.1186/s12883-022-02781-4)
Supplement: Supplementary file 1 — Additional file 1. Supplemental Table 1.1 Means, standard deviations, and medians for groups defined by control and migraine status, sub-setting by chronic vs episodic and medication overuse. Supplemental Table 1.2 Means, standard deviations, and medians for groups defined by control and migraine status, sub-setting by chronic vs episodic and medication overuse. Supplemental Table 1.3 Means, standard deviations, and medians for groups defined by control and migraine status, sub-setting by chronic vs episodic and medication overuse. [file 12883_2022_2781_MOESM1_ESM.zip › Supplemental Table 1.1.docx]

| **Supplemental Table 1.1 Means, standard deviations, and medians for groups defined by control and migraine status, sub-setting by chronic vs episodic and medication overuse** | | | | | |
| --- | --- | --- | --- | --- | --- |
| Variable | Controls | | Episodic migraine | | |
|  | n=7 | | n=7 | | |
|  | Mean (SD) | Median | Mean (SD) | Median | p value* |
| Monocytes (%) |  |  |  |  |  |
| Classical (CD16-CD14+) | 56.4 (26.6) | 39.9 | 79.3 (11.7) | 82.2 | 0.08 |
| Intermediate (CD16+CD14+) | 2.47 (1.91) | 1.86 | 6.16 (6.26) | 4.24 | 0.1 |
| Nonclassical (CD16+CD14-) | 25.5 (18.6) | 20.3 | 9.34 (8.87) | 7.23 | 0.1 |
| T cells (%) |  |  |  |  |  |
| CD4+ | 72.5 (9.58) | 69 | 66.9 (6.65) | 67 | 0.4 |
| CD8+ | 23.4 (5.7) | 26.2 | 22.1 (5.71) | 22.2 | 0.9 |
| CD4/CD8 | 3.37 (1.37) | 3.14 | 3.16 (0.70) | 3.3 | 0.8 |
| CD18(MFI) CD4 | 701 (207) | 601 | 663 (140) | 640 | 0.8 |
| CD18(MFI) CD8 | 872 (269) | 827 | 851 (316) | 775 | 0.8 |
| CD49(MFI) CD4 | 1224 (199) | 1226 | 1468 (271) | 1547 | 0.047 |
| CD49(MFI) CD8 | 1512 (266) | 1560 | 1555 (191) | 1612 | 1 |
| CD36 | 224 (170) | 178 | 359 (167) | 375 | 0.4 |
| CD4+CD25+ | 8.64 (2.98) | 7.61 | 5.56 (1.16) | 5.08 | 0.08 |

*p values in this table are based on comparisons with the controls based on the nonparametric Wilcoxon signed rank test (exact)
