# Supplementary material for: Leukocyte inflammatory phenotype and function in migraine patients compared with matched non-migraine volunteers: a pilot study
Source: BMC Neurol. 2022 Jul 27;22:278. doi: 10.1186/s12883-022-02781-4 (PMC9327171; doi:10.1186/s12883-022-02781-4)
Supplement: Supplementary file 1 — Additional file 1. Supplemental Table 1.1 Means, standard deviations, and medians for groups defined by control and migraine status, sub-setting by chronic vs episodic and medication overuse. Supplemental Table 1.2 Means, standard deviations, and medians for groups defined by control and migraine status, sub-setting by chronic vs episodic and medication overuse. Supplemental Table 1.3 Means, standard deviations, and medians for groups defined by control and migraine status, sub-setting by chronic vs episodic and medication overuse. [file 12883_2022_2781_MOESM1_ESM.zip › Supplemental Table 1.2.docx]

| **Supplemental Table 1.2 Means, standard deviations, and medians for groups defined by control and migraine status, sub-setting by chronic vs episodic and medication overuse** | | | | | |
| --- | --- | --- | --- | --- | --- |
| Variable | Controls | | Chronic migraine | | |
|  | n=8 | | n=8 | | |
|  | Mean (SD) | Median | Mean (SD) | Median | p value |
| Monocytes (%) |  |  |  |  |  |
| Classical (CD16-CD14+) | 44.1 (28.9) | 52.5 | 66.3 (21.2) | 76.8 | 0.25 |
| Intermediate (CD16+CD14+) | 2.02 (1.15) | 2.06 | 2.94 (2.92) | 2.53 | 0.6 |
| Nonclassical (CD16+CD14-) | 41.2 (22.1) | 35.8 | 14.2 (11.8) | 10.8 | 0.04 |
| T cells (%) |  |  |  |  |  |
| CD4+ | 68.8 (8.56) | 64.2 | 61.6 (2.53) | 62 | 0.04 |
| CD8+ | 19.1 (9.37) | 17.2 | 21.1 (7.92) | 22.3 | 0.1 |
| CD4/CD8 | 4.60 (2.52) | 4.54 | 2.48 (0.67) | 2.14 | 0.04 |
| CD18(MFI) CD4 | 763 (74.3) | 751 | 580 (125) | 557 | 0.04 |
| CD18(MFI) CD8 | 970 (217) | 1006 | 924 (239) | 948 | 0.02 |
| CD49(MFI) CD4 | 1266 (283) | 1358 | 956 (174) | 988 | 0.05 |
| CD49(MFI) CD8 | 1429 (377) | 1239 | 1127 (303) | 1139 | 0.2 |
| CD36 | 463 (274) | 459 | 195 (186) | 154 | 0.04 |
| CD4+CD25+ | 7.99 (1.67) | 8.02 | 5.74 (1.58) | 5.54 | 0.016 |
| *p values in this table are based on comparisons with the controls based on the nonparametric Wilcoxon signed rank test (exact) | | | | | |
