# Supplementary material for: Leukocyte inflammatory phenotype and function in migraine patients compared with matched non-migraine volunteers: a pilot study
Source: BMC Neurol. 2022 Jul 27;22:278. doi: 10.1186/s12883-022-02781-4 (PMC9327171; doi:10.1186/s12883-022-02781-4)
Supplement: Supplementary file 1 — Additional file 1. Supplemental Table 1.1 Means, standard deviations, and medians for groups defined by control and migraine status, sub-setting by chronic vs episodic and medication overuse. Supplemental Table 1.2 Means, standard deviations, and medians for groups defined by control and migraine status, sub-setting by chronic vs episodic and medication overuse. Supplemental Table 1.3 Means, standard deviations, and medians for groups defined by control and migraine status, sub-setting by chronic vs episodic and medication overuse. [file 12883_2022_2781_MOESM1_ESM.zip › Supplemental Table 1.3.docx]

| **Supplemental Table 1.3 Means, standard deviations, and medians for groups defined by control and migraine status, sub-setting by chronic vs episodic and medication overuse** | | | | | |
| --- | --- | --- | --- | --- | --- |
| Variable | Controls | | Medication overuse | | |
|  | n=8 | | n= 8 | | |
|  | Mean (SD) | Median | Mean (SD) | Median | p value* |
| Monocytes (%) |  |  |  |  |  |
| Classical (CD16-CD14+) | 50.9 (28.9) | 48.8 | 74.1 (22.6) | 84.5 | 0.25 |
| Intermediate (CD16+CD14+) | 2.41 (1.98) | 2.3 | 2.39 (2.41) | 1.68 | 0.9 |
| Nonclassical (CD16+CD14-) | 28.3 (17.3) | 31.4 | 9.44 (12.2) | 5.88 | 0.08 |
| T cells (%) |  |  |  |  |  |
| CD4+ | 70.6 (11.2) | 64 | 63.5 (6.38) | 63.5 | 0.25 |
| CD8+ | 22.1 (8.39) | 24.4 | 25.2 (6.15) | 24.4 | 0.5 |
| CD4/CD8 | 3.80 (2.00) | 2.95 | 2.66 (0.72) | 2.52 | 0.3 |
| CD18(MFI) CD4 | 712 (122) | 738 | 573 (111) | 569 | 0.1 |
| CD18(MFI) CD8 | 919 (232) | 946 | 731 (210) | 739 | 0.1 |
| CD49(MFI) CD4 | 1206 (234) | 1251 | 1307 (322) | 1272 | 0.5 |
| CD49(MFI) CD8 | 1489 (282) | 1551 | 1365 (368) | 1424 | 0.5 |
| CD36 | 347 (247) | 219 | 247 (155) | 238 | 0.4 |
| CD4+CD25+ | 8.23 (2.93) | 7.65 | 5.47 (1.66) | 5 | 0.04 |
| *p values in this table are based on comparisons with the controls based on the nonparametric Wilcoxon signed rank test (exact) | | | | | |
