# Supplementary material for: Leukocyte inflammatory phenotype and function in migraine patients compared with matched non-migraine volunteers: a pilot study
Source: BMC Neurol. 2022 Jul 27;22:278. doi: 10.1186/s12883-022-02781-4 (PMC9327171; doi:10.1186/s12883-022-02781-4)
Supplement: Supplementary file 2 — Additional file 2. Supplemental Table 2. Differences between migraines and controls adjusted for MOH and Chronic Migraine [file 12883_2022_2781_MOESM2_ESM.docx]

| **Supplemental Table 2. Differences between migraines and controls adjusted for MOH and Chronic Migraine** | | | | | | | |
| --- | --- | --- | --- | --- | --- | --- | --- |
| Variable | Unadjusted Mean difference (95% CI)^a^ | | Adjusted Mean difference  (95% CI)^b^ | |  |  |  |
|  | n=30 | | n=30 | |  |  |  |
| Monocytes |  |  |  |  |  |  |  |
| Classical (CD16-CD14+) | 22.48 | (6.34, 38.6) | 26.59 | (3.04, 50.12) |  |  |  |
| Intermediate (CD16+CD14+) | 2.21 | (-0.18, 4.61) | 6.59 | (3.63, 9.55) |  |  |  |
| Nonclassical (CD16+CD14-) | -21.9 | (-33.6, -10.2) | -21.65 | (-38.88, -4.42) |  |  |  |
| T cells (%) |  |  |  |  |  |  |  |
| CD4+ | -6.53 | (-11.45, -1.61) | -2.82 | (-10.17, 4.52) |  |  |  |
| CD8+ | 3.23 | (-1.16, 8.14) | -0.17 | (-7.29, 6.94) |  |  |  |
| CD4/CD8 | -1.22 | (-2.31, -0.14) | -0.67 | (-2.3, 0.93) |  |  |  |
| CD18(MFI) CD4 | -115 | (-214, -17.8) | -12.5 | (-150, 125) |  |  |  |
| CD18(MFI) CD8 | -152 | (-321, 16.5) | -17.3 | (-260, 226) |  |  |  |
| CD49(MFI) CD4 | -51.7 | (-255, 152) | 100 | (-113, 313) |  |  |  |
| CD49(MFI) CD8 | -141 | (-367, 84.8) | 57.1 | (-239, 352) |  |  |  |
| CD36 | -76.8 | (-235, 76.1) | 46.4 | (-176, 269) |  |  |  |
| CD4+CD25+ | -2.63 | (-3.94, -1.33) | -2.51 | (-4.46, -0.57) |  |  |  |
| ^a^ Analysis uses a linear hierarchical model with random effects of paired controls/patients | | | | | | | |
| ^b^ Adjusted models control for chronic (vs. episodic) migraine and presence of medication overuse headache | | | | | | | |
